# Shear flow promotes bacterial growth and shapes spatial gradients by rapidly replenishing scarce nutrients

**DOI:** 10.1128/mbio.03446-25

**Published:** 2026-02-27

**Authors:** Gilberto C. Padron, Sizhe Chen, Anuradha Sharma, Zil Modi, Matthias D. Koch, Joseph E. Sanfilippo

**Affiliations:** 1 Department of Biochemistry, University of Illinois at Urbana-Champaign 14589, Urbana, Illinois, USA; 2 Department of Biology, Texas A&M University 14736, College Station, Texas, USA; Yale University School of Medicine, New Haven, Connecticut, USA

**Keywords:** fluid flow, bacteria, microfluidics, nutrients, *Pseudomonas aeruginosa*

## Abstract

In laboratory settings, bacteria grow in static culture with more nutrients than they require. However, bacteria in nature experience flowing environments that are nutrient-limited. Using microfluidics and single-cell imaging, we discover that shear flow promotes growth of the human pathogens *Pseudomonas aeruginosa* and *Vibrio cholerae* at surprisingly low nutrient concentrations. In static environments, cells require high nutrient concentrations as they steadily consume non-renewable resources. In slower-flowing environments, cells grow and deplete nutrients, which generates spatial gradient profiles. In faster-flowing environments, cells grow robustly and form microcolonies even at very low concentrations due to rapid nutrient replenishment. By precisely delivering nutrients using microfluidics, we learned that cells in flow can grow on glucose concentrations 1,000 times lower than those observed in typical laboratory experiments. The ultralow glucose concentrations sufficient for growth in flow closely align with the affinity of bacterial glucose transporters, suggesting that bacteria have evolved in flowing environments with scarce nutrients. Collectively, our results emphasize the limits of traditional culturing approaches and highlight how shear flow can promote bacterial growth and shape spatial gradients.

**IMPORTANCE:**

While bacteria in nature experience flow, laboratory conditions typically omit flow. Additionally, bacteria in nature are often nutrient-limited, but laboratory conditions contain excess nutrients. Here, we use microfluidic technology to determine how flow impacts growth of a bacterial pathogen under nutrient limitation. We discover that flow sustains growth at glucose concentrations 1,000 times lower than traditionally observed. In traditional experiments, bacteria grow on a high concentration of a non-renewable resource. In our microfluidic experiments, bacteria can grow on surprisingly low concentrations of resources if they are renewed by flow. Our results emphasize the need to study bacteria in realistic contexts and suggest that scientists should rethink how cells experience nutrient limitation in nature.

## INTRODUCTION

Many natural environments are constantly changing due to fluid flow. However, experimental systems typically simplify nature and fail to include flow-driven dynamics. Recent studies using microfluidics have revealed how studying bacteria in flow can shift our understanding of biological processes (1–3). For example, the shear force associated with flow has the counterintuitive effect of enhancing bacterial adhesion (4–7). In addition, flow physically orients bacteria to move upstream on surfaces (8–10), and flow promotes surface colonization by increasing daughter cell attachment (11, 12). Based on these reports, it now appears essential to include flow in our study of bacterial processes to fully understand how bacteria survive in nature.

Biological flow responses fall into two categories: responses to the shear force associated with flow (2, 4) and responses to flow-driven chemical transport (13, 14). While the impact of shear force on bacterial cells involves stretching or bending (15), flow-driven chemical transport can replenish or wash away small molecules. Logically, the transport of small molecules by flow can amplify or inhibit bacterial processes that depend on local chemical concentrations. For example, flow can amplify the impact of antimicrobials by replenishing molecules faster than cells can remove them (14, 16, 17). In contrast, flow can inhibit quorum sensing by washing away autoinducers faster than they diffuse back to cells (18, 19). In both of these examples, including flow in experiments dramatically changed the results and the interpretation of how bacteria function in natural environments. Thus, using microfluidics to test the impact of flow-driven transport offers a great opportunity to better understand how bacteria interact with their chemical environment.

Bacteria require an adequate supply of nutrients to grow (20, 21). In laboratory experiments, bacteria are typically grown in culture media with an excess of nutrients (22, 23). While complex media allow for robust growth of diverse bacterial species, the use of minimal media allows for precise characterization of nutrient requirements. Minimal media typically consist of a few defined ingredients: water, a carbon source, and various salts, which provide elements necessary for growth (24). The presumed advantage of minimal media is that all added ingredients are known. By limiting one ingredient at a time, researchers have determined how specific nutrients impact bacterial growth in static culture (25–27). However, it remains unclear how nutrient limitation impacts growth in flowing conditions. Based on the success of studying other chemical transport processes under flow (14, 17, 19, 28, 29), we hypothesize that combining flow and nutrient limitation should yield important new insights.

Using microfluidics, we discover that flow enhances growth of *Pseudomonas aeruginosa* and *Vibrio cholerae* in nutrient-limited conditions. First, we establish the nutrient concentrations required for growth in traditional batch culture. Second, our results reveal that cells in flow can grow and form microcolonies at surprisingly low nutrient concentrations. Third, we combine simulations and microfluidic experiments to quantitatively determine how flow modulates spatial and temporal growth gradients. Fourth, we demonstrate that *P. aeruginosa* cells in flow can grow with glucose concentrations 1,000 times lower than traditionally observed. Notably, our finding that bacteria can grow on low micromolar glucose levels closely matches the known affinity of bacterial glucose transporters (30, 31), suggesting that bacteria have evolved in nutrient-depleted flowing environments.** **

## RESULTS

**Fig 1:**
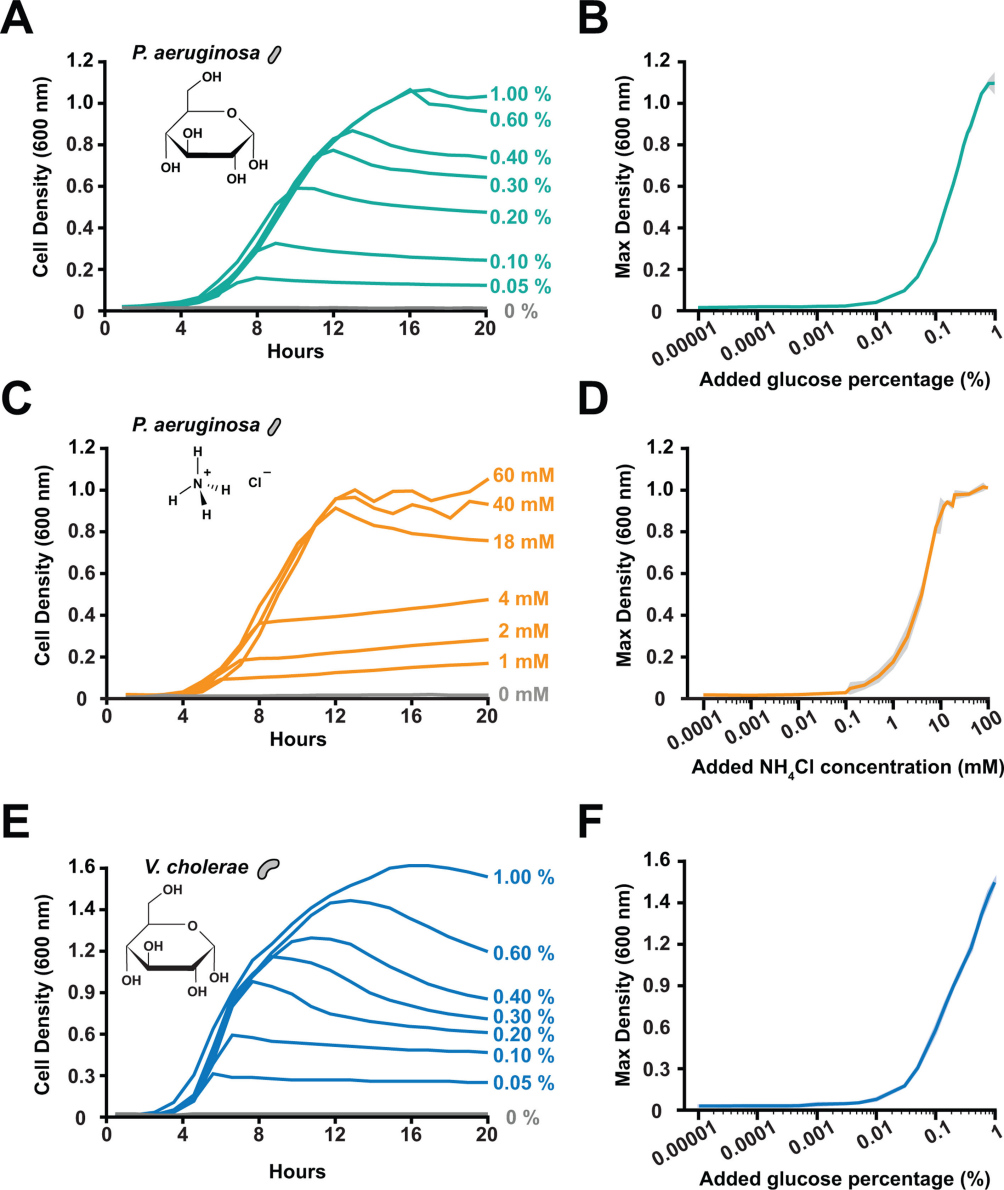
Bacteria require high nutrient concentrations for batch culture growth. (**A**) Growth curves of *P. aeruginosa* cells in M9 minimal medium with varying glucose concentrations. Cell density measured by optical density at 600 nm. While increasing glucose impacted maximal culture density, it did not impact growth rate. (**B**) Maximum culture density of *P. aeruginosa* cells grown in varying glucose concentrations. Lines and shading show the average and standard deviation of three biological replicates. (**C**) Growth curves of *P. aeruginosa* cells in M9 minimal medium with varying ammonium chloride concentrations. Cell density measured by optical density at 600 nm. While increasing ammonium chloride impacted maximal culture density, it did not impact growth rate. (**D**) Maximum culture density of *P. aeruginosa* cells grown in varying ammonium chloride concentrations. Lines and shading show the average and standard deviation of three biological replicates. (**E**) Growth curves of *V. cholerae* cells in M9 minimal medium with varying glucose concentrations. Cell density measured by optical density at 600 nm. While increasing glucose impacted maximal culture density, it did not impact growth rate. (**F**) Maximum culture density of *V. cholerae* cells grown in varying glucose concentrations. Lines and shading show the average and standard deviation of three biological replicates. All shaded error bars are gray, semi-transparent overlays. Due to very small standard deviations, some of the shading is difficult to notice.

To begin our study of nutrient limitation in *P. aeruginosa*, we quantified growth in batch culture. We used M9 minimal medium with glucose as the sole added carbon source and ammonium chloride as the sole added nitrogen source. To examine how glucose concentration impacts growth, we varied the glucose concentration while keeping all other nutrients constant. From 0.05% to 1% glucose, the growth rate for the early portion of the experiment remained similar (Fig. 1A; Fig. S1). However, higher glucose concentrations led to higher maximal culture density, while lower concentrations led to lower maximal density (Fig. 1A). By varying glucose concentrations over many orders of magnitude, we observed that approximately 0.01% glucose was required to support detectable growth in batch culture (Fig. 1B). Similarly, by varying the ammonium chloride concentration while keeping all other nutrients constant, we found that approximately 0.1 mM ammonium chloride was required to support detectable growth in batch culture (Fig. 1C and D). To broaden our findings to other bacteria, we repeated our glucose experiments with *Vibrio cholerae* and observed that approximately 0.01% glucose was required for detectable batch culture growth (Fig. 1E and F). Together, these experiments quantitatively establish the minimum nutrient concentrations required to support *P. aeruginosa* and *V. cholerae* growth in batch culture.

**Fig 2:**
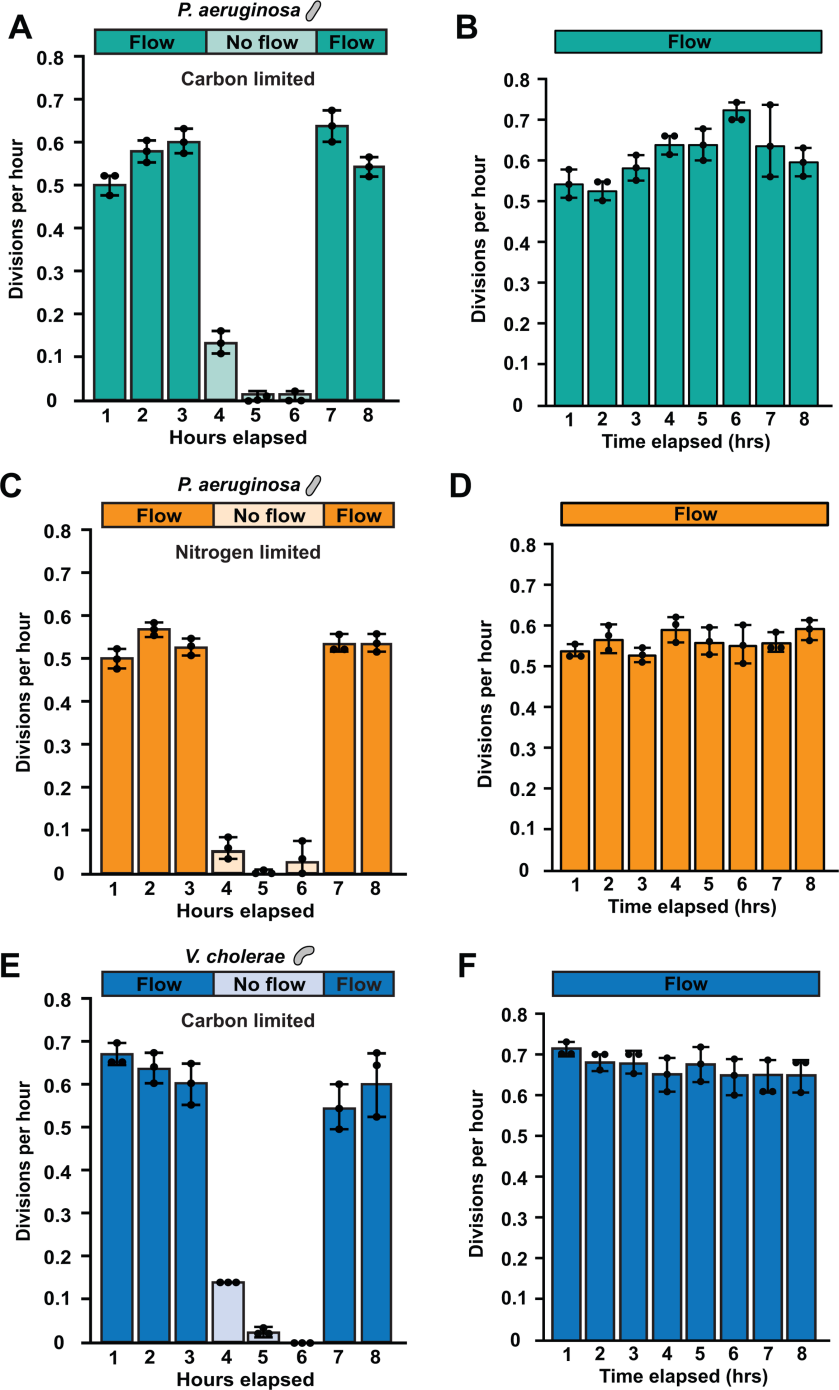
Flow is required for growth with extremely low nutrient concentrations. Quantification of cell division per hour under variable flow (**A**) or constant flow (**B**) carbon-limited *P. aeruginosa*, variable flow (**C**) or constant flow (**D**) nitrogen-limited *P. aeruginosa*, and variable flow (**E**) or constant flow (**F**) carbon-limited *V. cholerae* experiments. Carbon-limited indicates M9 minimal medium without an added carbon source, and nitrogen-limited indicates M9 minimal medium without an added nitrogen source. When flow was on, cells could grow in both carbon- and nitrogen-limited regimes. When flow stopped, cells were quickly unable to grow. Quantification shows the average and standard deviation of three biological replicates. For each biological replicate, 50 cells were chosen at random for quantification.

To examine nutrient limitation with single-cell resolution in flow, we imaged *P. aeruginosa* and *V. cholerae* cells in microfluidic devices (Fig. S2). Aiming to determine the minimum glucose concentration required to support bacterial growth in flow, we exposed *P. aeruginosa* cells to M9 minimal medium with varying levels of glucose. For these experiments, we used a shear rate of 800 s^−1^, which is similar to the flow present in the bloodstream and urinary tract (32, 33). Surprisingly, our negative control with no added glucose grew robustly (Fig. S3). Cells exposed to M9 minimal medium with no added glucose grew consistently over an 8 h period at a shear rate of 800 s^−1^ (Fig. 2; Fig. S3). In all our experiments, we represent the flow intensity using shear rate, which describes the flow rate and channel dimensions. Inspired by our observation of growth with no added carbon source, we measured whether *P. aeruginosa* cells could grow with no added nitrogen source. Similarly, cells exposed to M9 minimal medium with no added ammonium chloride grew consistently over an 8 h period at 800 s^−1^ flow (Fig. 2; Fig. S3 and S4). Broadening the significance of our results, we also observed growth of *V. cholerae* in flow with M9 minimal medium with no added glucose (Fig. 2F). We interpreted these results to indicate that bacteria in flow were capable of growing on carbon and nitrogen contaminants present in our media. Based on these results, we will use the term “carbon limited” to indicate no added carbon source and “nitrogen limited” to indicate no added nitrogen source.

Does flow promote bacterial growth in nutrient-limited environments? As growth without added carbon or nitrogen sources was observed in microfluidic devices (Fig. 2; Fig. S3 and S4) but not in batch culture (Fig. 1), we hypothesized that flow promotes growth in nutrient-limited regimes. By turning flow on and off in microfluidic devices, we tested the flow dependency of *P. aeruginosa* and *V. cholerae* growth in nutrient-limited conditions. In conditions with flow, both species grew in nutrient-limited conditions (Fig. 2). However, when flow stopped, both species stopped growing (Fig. 2). Growth resumed when we restarted flow, indicating that flow promotes *P. aeruginosa* and *V. cholerae* growth in nutrient-limited environments. These findings support the hypothesis that flow replenishes nutrients fast enough that cells can continuously utilize them, allowing for growth at lower nutrient concentrations than previously observed.

**Fig 3:**
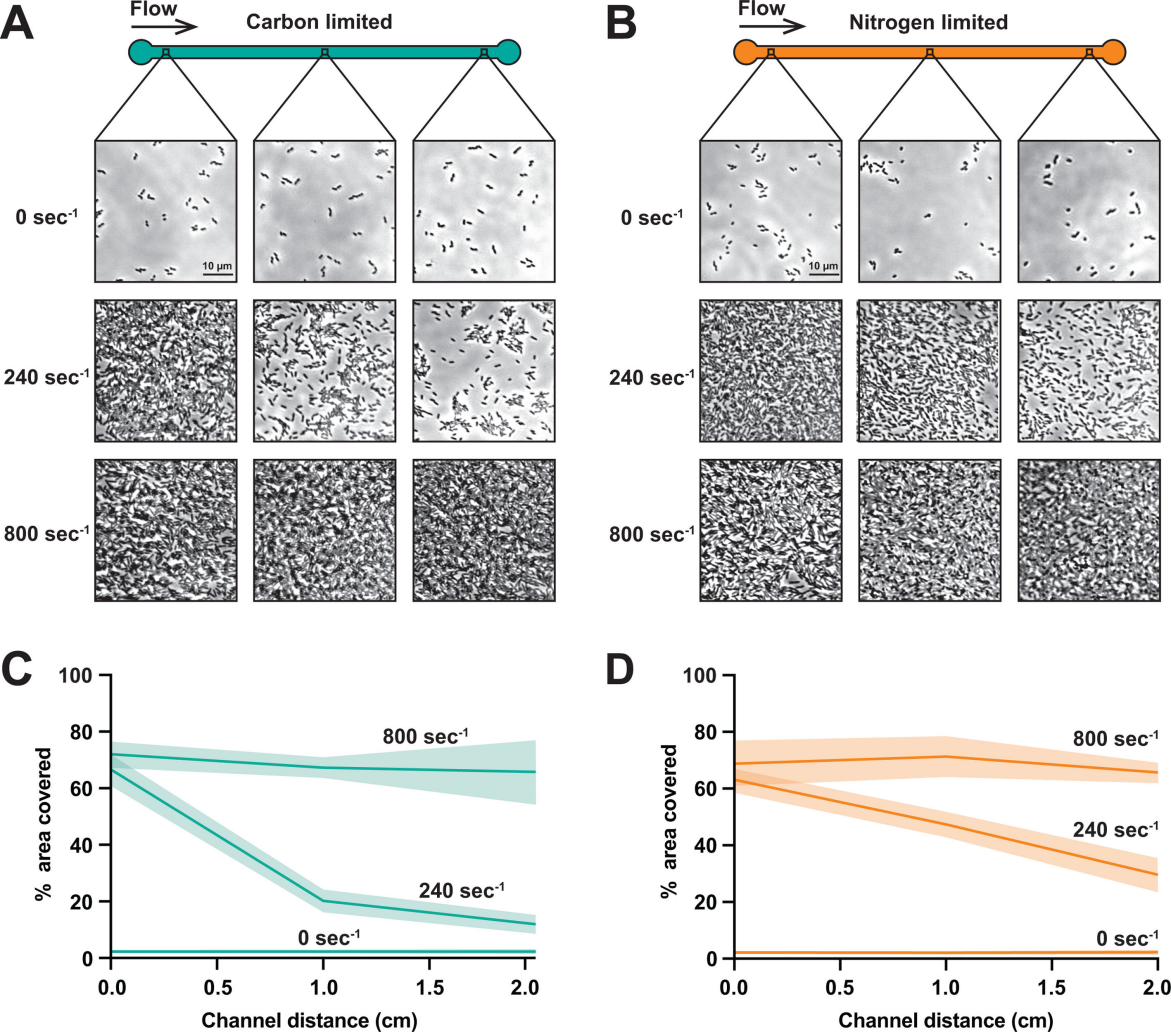
Flow promotes microcolony development in nutrient-limited conditions. Visual representation of the 2 cm microfluidic device channel and phase-contrast images of *P. aeruginosa* under the shear rates of 0 s^−1^, 240 s^−1^, or 800 s^−1^ in the carbon-limited condition (**A**) or the nitrogen-limited condition (**B**). Brightness levels of the phase-contrast images were uniformly adjusted for clarity. The black boxes in the 2 cm channel indicate the localization of where the phase-contrast images were captured, corresponding to the channel’s beginning, middle, and end. (**C**) Estimated percentage of area covered by the cells after 24 h in the carbon-limited condition. (**D**) The estimated percentage of area covered by the cells after 19 h in the nitrogen-limited condition. Quantification indicates the average and standard deviation of three biological replicates.

Does flow promote microcolony development in nutrient-limited environments? As bacterial biofilms are problematic in healthcare and industrial settings where nutrients are typically not provided, we used our microfluidic system to interrogate the impact of flow on the early *P. aeruginosa* biofilm development. Using microfluidic devices with a carbon-limited medium, we observed that cells without flow were unable to form microcolonies (Fig. 3A and C). When a carbon-limited medium was delivered at a shear rate of 240 s^−1^, we observed a spatial gradient, with robust microcolonies at the start of the channel and smaller microcolonies at the end of the channel (Fig. 3A and C). When we used a shear rate of 800 s^−1^, cells exposed to carbon-limited conditions developed robust microcolonies throughout the channel (Fig. 3A and C). Similarly, cells exposed to nitrogen-limited conditions were unable to form microcolonies without flow, formed spatial gradients at 240 s^−1^, and formed microcolonies throughout the channel at 800 s^−1^ (Fig. 3B and D). Together, our results demonstrate that flow promotes microcolony development in nutrient-limited regimes.

To quantitatively understand how flow promotes single-cell growth and microcolony development in nutrient-limited conditions, we simulated *P. aeruginosa* cells during a transition from flow to no flow (Fig. S5). Our simulations included three parameters: flow, diffusion, and nutrient removal. By simulating microfluidic channels with flow, we established a baseline nutrient concentration. Then, we stopped flow and quantified how quickly nutrients were depleted from the simulated channel (Fig. S5). For a carbon source the size of glucose, our simulations estimate that approximately 90% of molecules are removed within 30 min of stopping flow (Fig. S5). Similarly, for a nitrogen source the size of ammonium chloride, our simulations estimate that approximately 90% of molecules are removed within 15 min of stopping flow (Fig. S5). Thus, our simulations generate the testable prediction that available carbon and nitrogen will be depleted within min after flow stops.

Guided by our simulations, we experimentally tested the impact of stopping flow on cell growth. To precisely measure growth of cells on short timescales, we quantified the length of cells exposed to constant flow or flow that had just been stopped (Fig. S5). As we were interested in observing cell growth at timescales where a cell division is not an optimal metric, we instead focused on elongation, or the accumulation of mass as a cell lengthens prior to its division. Cells in carbon-limited or nitrogen-limited conditions with constant flow elongated at a constant rate (Fig. S5). In contrast, cells in carbon-limited conditions stopped elongating approximately 30 min after flow stopped (Fig. S5). Similarly, cells in nitrogen-limited conditions stopped elongating approximately 10 min after flow stopped (Fig. S5). Our experiments validate our simulations (Fig. S5), demonstrate that cells rapidly stop growing when flow stops, and further support that flow promotes growth by replenishing nutrients fast enough that cells can continuously utilize them.

**Fig 4:**
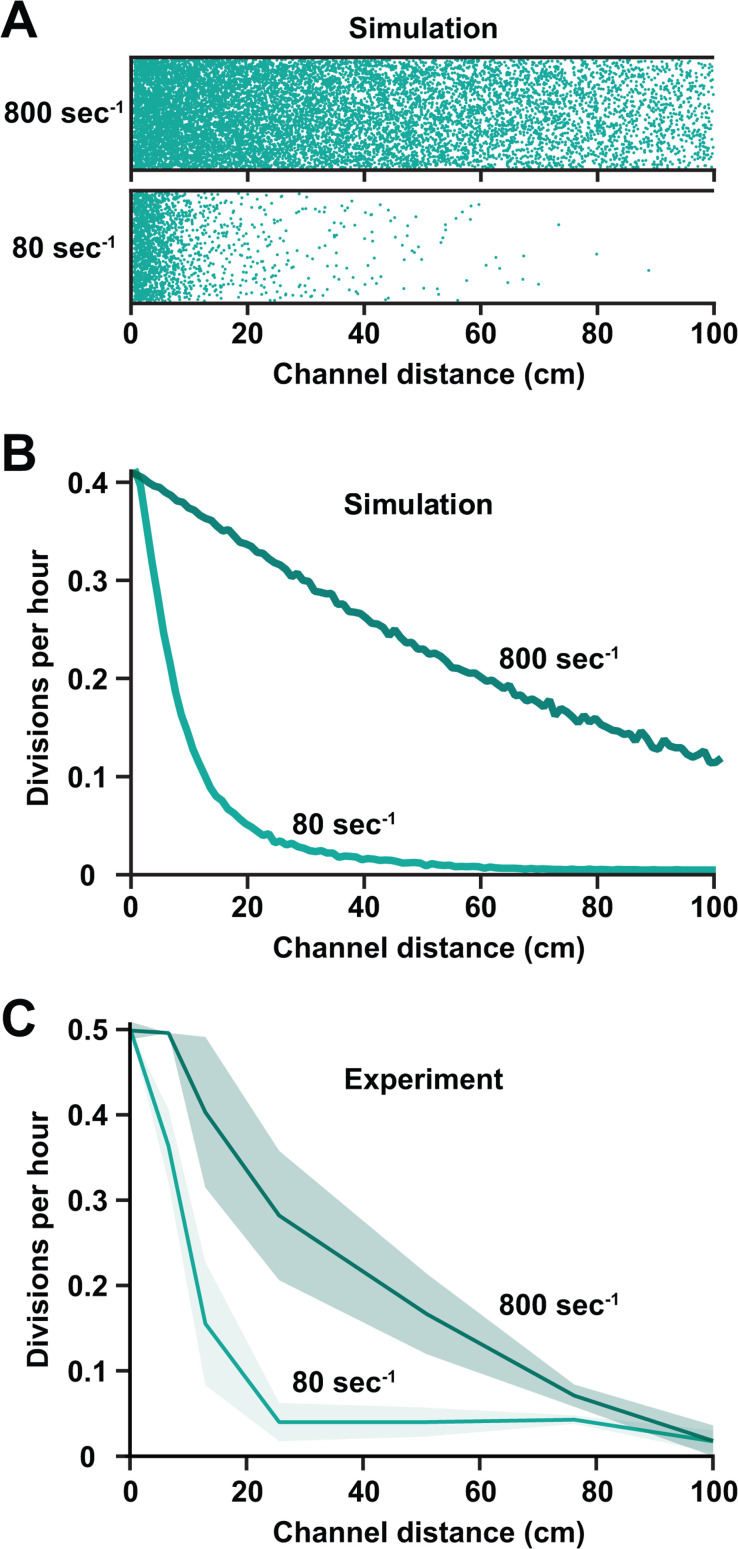
Flow shapes spatial gradients of nutrient availability and growth. (**A**) Visual representation of glucose molecules across simulated 1 m microfluidic channels at different shear rates. Teal dots represent individual glucose molecules experiencing diffusion in all directions, flow from left to right, and removal when they hit the bottom of the channel. (**B**) Simulated growth gradients based on simulated glucose profiles and the relationship between glucose concentration and growth rate. Faster flow increases the glucose concentration and the growth rate. (**C**) Experimental growth gradients showing the relationship between flow, channel length, and growth rate. Lines and shading show the average and standard deviation of three biological replicates. For each biological replicate, 30 cells were chosen at random for quantification. The experimental results in panel C closely match the simulated results in panel B.

How does shear rate impact growth in nutrient-limited environments? As increasing shear rate promoted microcolony development (Fig. 3), we reasoned that shear rate is important due to its role in facilitating nutrient replenishment. To quantitatively test our reasoning, we simulated a carbon-limited environment in long microfluidic channels with different shear rates. At a low shear rate (80 s^−1^), short spatial gradients form as molecules are removed before they reach the end of the channel (Fig. 4A and B). In contrast, at a high shear rate (800 s^−1^), long spatial gradients form as flow transports molecules deeper into the channel before they can be removed (Fig. 4A and B). While performing these simulations, we realized that preferential growth at the beginning of the channel may alter the spatial gradient over time. By adding a feedback loop to our simulation where increased growth leads to an increase in the uptake rate (because cells grow and divide), our simulations revealed that spatial gradients become steeper and shift forward over time (Fig. S6). Thus, our simulations predict that shear rate has an important role in shaping nutrient gradients across bacterial populations.

To experimentally test the role of shear rate, we measured bacterial growth across long microfluidic channels. Using 1 m-long channels (Fig. S2), we quantified growth of *P. aeruginosa* cells in a carbon-limited environment exposed to a low shear rate (80 s^−1^). Comparable to the spatial gradients we observed in microcolony development (Fig. 3), cells at the start of the channel grew well, and growth decreased quickly as a function of channel length (Fig. 4C). At a higher shear rate (800 s^−1^), we observed that cells grew better and exhibited longer spatial gradients (Fig. 4C). The observed spatial gradients for both shear rates were very similar to our simulated spatial gradients (Fig. 4B). Analogous shear rate experiments in nitrogen-limited conditions revealed similar gradients (Fig. S7), indicating that shear rate is generally important during nutrient limitation. Further supporting our simulations (Fig. S6), we observed that our spatial gradients became steeper and shifted forward over time (Fig. S8). Together, our results establish that shear rate has an important role in delivering nutrients into populations, overcoming nutrient limitation, and shaping spatial gradients.

**Fig 5:**
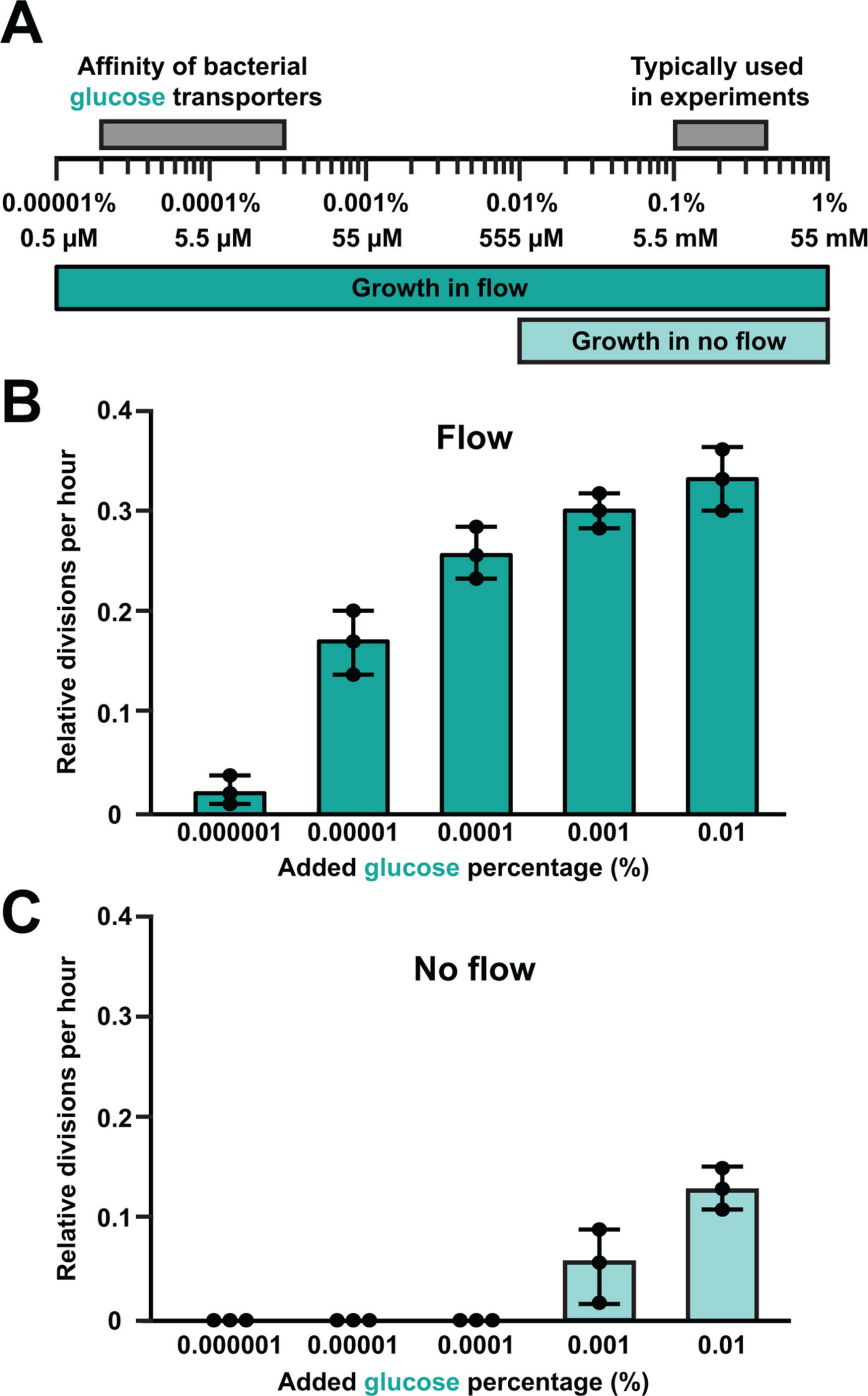
Bacteria grow with 1,000 times lower glucose concentrations in flow. (**A**) Representation of glucose concentrations required for growth in flow and in no flow. For comparison, the glucose concentrations typically used in experiments and the affinity of bacterial glucose transporters were included (30, 31). Cell divisions per hour under flow at a shear rate of 800 s^−1^ (**B**) or no flow (**C**) using conditioned M9 minimal medium with various concentrations of added glucose. M9 minimal medium was conditioned by cells to remove contaminant carbon sources. Quantification represents the average and standard deviation of three biological replicates. For each biological replicate, 30 cells were chosen at random for quantification. Experiments were conducted in microfluidic devices with or without flow for direct comparison of results.

What is the minimum glucose concentration required for growth in flow? As *P. aeruginosa* cells grow in flow with no added carbon source (Fig. 2; Fig. S3 and S4), we hypothesized that cell growth requires very low available carbon. However, as cells were growing on a carbon contaminant (Fig. S3), we were unable to quantify how much carbon is required for growth in flow. To overcome this challenge, we flowed our media through microfluidic channels filled with cells at a very high density. At high density, cells depleted the contaminating carbon source and were unable to grow (Fig. S7). By collecting and filtering the conditioned media, we precisely determined how much carbon is required for growth in flow (Fig. 5). While conditioned media alone could not support *P. aeruginosa* growth in a new channel, conditioned media with 0.00001% glucose added was capable of supporting growth in flow (Fig. 5B). In contrast, 0.01% was required to support the same growth in channels without flow (Fig. 5C). Thus, our results establish that bacterial cells can grow with 1,000 times lower glucose concentrations in flowing conditions.

## DISCUSSION

Here, we report that shear flow promotes bacterial growth and shapes population gradients by rapidly replenishing ultralow nutrient concentrations. Using M9 minimal medium, we demonstrated that cells in batch culture require 0.01% glucose and 0.1 mM ammonium chloride for growth (Fig. 1). In contrast, our microfluidic experiments revealed that cells can grow (Fig. 2) and form microcolonies (Fig. 3) on contaminant levels of carbon and nitrogen when delivered by flow. Combining simulations and long microfluidic channels, we established how shear rate patterns spatial gradients of nutrients and growth across bacterial populations (Fig. 4). Finally, we discovered that 0.00001% glucose could support the same amount of growth in flow as 0.01% glucose could support without flow (Fig. 5). Together, our results highlight how the interplay between the physical and chemical environment impacts bacterial physiology in nutrient-limited regimes.

How do bacteria experience nutrient limitation in nature? While it is difficult to conclusively know how nutrient limitation impacts bacteria in nature, extensive laboratory experimentation using batch culture gives some clues. In batch culture, cells are typically provided with excess nutrients that support growth for many generations. During this phase of exponential growth, cells replicate quickly as they are not limited for nutrients. Eventually, the pool of available nutrients depletes, and the rate of growth slows. At this point, cells enter a nutrient-limited regime. However, as this shift occurs rapidly in batch culture, it is technically challenging to quantify the nutrient concentration during the transition to nutrient limitation. In nature, bacteria likely spend most of their lives transitioning in and out of nutrient limitation (34). For example, bacteria in freshwater (35), marine (36), and host (37) environments are often subjected to nutrient-limited flowing environments. Here, we used microfluidics to overcome this challenge and illustrate that bacteria in flow can robustly grow at concentrations that we expected to be limiting. Our unexpected results call for a reevaluation of bacterial nutrient limitation and emphasize that simplified laboratory conditions poorly reflect complex natural environments.

While interpreting our results, we wondered how our microfluidic devices compared to bacterial chemostats. Conceptually, both replenish nutrients to sustain bacterial growth. In our microfluidic devices, the total volume inside the channel is approximately 0.5 µL. Based on the host-relevant shear rate we used, the time required to replace all the media in the channel is ~3 s. In contrast, a typical chemostat has a volume greater than 100 mL. Based on typical chemostat parameters (38), the time required to replace all the media in a chemostat is at least multiple hours. As our experiments replenish the media ~1,000 times faster than a chemostat, we expect cells in our devices to grow on nutrient concentrations much lower than those found in a chemostat. Consistent with that prediction, glucose-limited bacterial chemostat experiments typically have ~0.01% glucose (38–42), which closely matches the glucose concentration required for growth in our no-flow microfluidic experiments (Fig. 5C). In contrast, our microfluidic experiments with flow facilitate growth at 0.00001% glucose (Fig. 5B). Given these major differences between a microfluidic device and a chemostat in both the replenishment time and glucose concentrations, being 1,000 times faster and 1,000 times lower, respectively, we conclude that our microfluidic experiments are very different from previous chemostat experiments.

Our discovery that bacteria in flow can grow on extremely low glucose concentrations suggests that bacteria have evolved on the edge of nutrient limitation. To explore this idea further, we compared the glucose concentrations sufficient for growth in flow with the affinity of bacterial glucose transporters. Glucose enters the periplasm of gram-negative bacteria through various non-specific outer membrane porins (30). Periplasmic glucose is then transported into the cytoplasm through specific inner membrane transporters (43–46). In *P. aeruginosa*, the *K*_*m*_ for glucose transporters is approximately 8 µM–10 µM (31, 45, 47). In *Escherichia coli*, the *K*_*m*_ for glucose transporters is approximately 5 µM–20 µM (30, 48–51). Strikingly, these values closely align with each other and with the concentrations sufficient to support growth under flow (Fig. 5A). The alignment between species suggests that bacteria have evolved to scavenge limiting resources away from competitors. The alignment between transporter affinity and growth requirements in flow suggests that bacteria have evolved to scavenge low concentrations of nutrients to support growth. In support of this idea, chemostat experiments and mathematical modeling predict that low micromolar glucose concentrations should support growth of environmental isolates of bacteria and mediate competition between them (52, 53). Collectively, our results highlight how studying nutrient limitation in microfluidic devices can enhance our understanding of bacterial behavior and physiology.

How does flow-driven chemical transport impact bacteria? Fundamentally, transport is the process of moving something from one place to another. For bacteria in laboratory conditions, nutrient transport is typically driven by diffusion. In closed systems like batch culture, the supply of nutrients by diffusion continues until depletion. However, many natural environments are open systems where transport is facilitated by flow. In these situations, the supply of nutrients by flow is effectively never depleted. Thus, closed diffusion-driven systems require high nutrient concentrations to support many generations of growth, while open flow-driven systems can support many generations of growth on much lower nutrient concentrations. Similar to our findings, recent studies have provided evidence that flow has an important role in many biological processes that depend on small molecules (14, 16, 17, 19, 54, 55). For example, flow’s ability to transport autoinducers suppresses local quorum sensing and facilitates long-distance cell-cell communication (19). Additionally, flow’s ability to deliver antimicrobials faster than cells can neutralize them overcomes bacterial resistance (17). As flow plays a critical role in bacterial growth, cell-cell signaling (19), and antimicrobial resistance (17), it is now clear that experiments should include flow in order to capture the complexities of natural environments.

## MATERIALS AND METHODS

### Strains, plasmids, and growth conditions

Bacterial strains used in this study are WT PA14, a ∆*pilA* mutant of WT PA14 (6), and *V. cholerae* O1 El Tor (56). All bacterial strains used were plated on LB plates. All *P. aeruginosa* and *V. cholerae* cultures, unless explicitly stated, were grown at 37°C in M9 minimal medium (produced in-house) supplemented with 20% glucose (VWR Life Science) to a final dilution of 0.4% as the carbon source. M9 minimal medium was prepared using the Cold Spring Harbor protocol for M9 minimal medium preparation (57), freely available from their website. Modifications to the M9 minimal medium were made by excluding either glucose or ammonium chloride (VWR Life Science) depending on the experimental condition being tested. Glucose (0%–2%) and ammonium chloride (0 mM–200 mM) concentrations were then supplemented into the M9 minimal medium as necessary. M9 minimal medium was additionally supplemented with 1 M magnesium sulfate (Fisher Chemical), 1 M calcium chloride (Sigma-Aldrich), and adjusted to pH 7.4 using sodium hydroxide for a working solution.

### *P. aeruginosa* and *V. cholerae* growth curves

Overnight cultures of *P. aeruginosa* and *V. cholerae* were grown in M9 minimal medium as described above. 96-well plates (Avantor) were prepared with addition of glucose (0%–2%) and ammonium chloride (0 mM–200 mM). Total media volume was set to 200 µL to ensure a 1:100 dilution of overnight cells (2 µL). Cells were shaken in a plate reader (BioTek Synergy S1) at 37°C, and optical density measurements were made every hour for 24 h. Growth curves were derived directly from Gen5 plate reader software using an M9 no-cells control as a blank for background subtraction.

### Fabrication of microfluidic devices

Microfluidic devices were created and fabricated using soft lithography. Devices were designed on Illustrator (Adobe Creative Suite), and masks were printed by CAD/Art Services. Molds were produced using 100 mm silicon wafers (University Wafer) and were spin-coated using SU-8 3050 photoresist (MicroChem). Polydimethylsiloxane chips were plasma-treated for bonding onto 60 mm × 35 mm × 0.16 mm superslip micro cover glass (Ted Pella, Inc.). Devices used in concentration and flow-to-no-flow-to-flow experiments were designed as seven parallel channels, 500 µm wide × 50 µm tall × 2 cm long. Long-channel experiments were conducted in devices 500 µm wide × 50 µm tall × 1 m long. Both devices contained a single inlet and outlet per channel.

### Phase-contrast microscopy

Timelapse images were captured using a Nikon ECLIPSE Ti2-E inverted microscope using the stock NIS Elements interface. The microscope was equipped with a Nikon 40× Plan Ph2 0.95 NA objective, a Hamamatsu ORCA-Flash4.0 LT3 Digital CMOS Camera, and a Lumencor SOLA Light Engine LED source.

### *P. aeruginosa* and *V. cholerae* in microfluidic devices

Prior to loading bacterial cultures, outlets were hooked up with tubing for waste outflow (Brain Tree Scientific Polyethylene Tubing; ID 0.015” × OD 0.043”). *P. aeruginosa* and *V. cholerae* were loaded using 10 µL of mid-log (0.4 OD) culture back-diluted from overnight cultures 1:100. Experimental media was loaded into 5 mL syringes (BD) and hooked up using standard tubing over a 26-gauge × ½” hypodermic needle (Air-tite Products) to microfluidic device. Syringes were placed on a syringe pump (KD Scientific Legato 210), which was used to vary flow rate (0 µL/min–10 µL/min), which resulted in shear rates from 0 to 800 s^−1^. For 1 m microfluidic device experiments, mid-log (0.4 OD) culture was pre-loaded onto channel using a syringe pump. Five-milliliter syringes (BD) were loaded with 1 mL of back-diluted culture and loaded into a microfluidic device prior to imaging in a biosafety cabinet (Labconco Logic+). A syringe pump was used to load cells into the device at a shear rate of 400 s^−1^ for 10 min. Five minutes of loading was conducted at the inlet of the device and 5 min at the outlet to most approximately equilibrate the concentration of cells across the device. For some experiments, flow was stopped by turning the syringe pump off. All experiments with 2 cm-long devices were imaged 1 cm into the channel. For 1 m experiments, images were taken at 0.1, 6.25, 12.5, 25, 50, 75, and 97.5 cm distances.

### Quantification of *P. aeruginosa* and *V. cholerae* growth

All experiments, unless described explicitly, followed the same workflow. Timelapse videos were collected every 5 min for up to 8 h. Videos were then analyzed using FIJI (Fiji is just ImageJ) software. Thirty or fifty cells were picked at random per experimental replicate and tracked for the duration of the experiment. Divisions were attributed only when cells had fully replicated as “1” and incrementally increased by one per replication event (e.g. 1 cell divides into 2 [total: one division], 1 cell divides into 4 [total: two divisions] and so forth). Total cell divisions were then averaged for all 30 or 50 cells and divided by the length of the experiment to obtain divisions per hour. Divisions per hour were averaged across three biological replicates, and a standard deviation was obtained. In experiments where growth (as an accumulation of mass) was measured prior to division, we measured elongation. Elongation measures the lengthening of cells and represents an alternative metric to divisions, as we cannot measure elongation once a cell divides. Thirty cells were selected at random, and length was measured at the start and stop of flow. Relative cell length was quantified as the length of the cell prior to a division when compared to the starting length of the cell. Elongation was also averaged across three biological replicates, and a standard deviation was obtained. All growth experiments for *P. aeruginosa* were performed with cells lacking *pilA*, which prevented twitching motility and allowed for efficient cell tracking. Growth experiments for *V. cholerae* were conducted by monitoring cells to ensure adherence for post-experiment quantification during each experiment’s entire runtime.

### Quantification of *P. aeruginosa* microcolony formation in microfluidic devices

Surface colonization was measured in carbon- or nitrogen-limited M9 minimal medium over 24 h. All growth experiments for *P. aeruginosa* were performed with cells lacking *pilA*. After seeding the microfluidic devices with mid-log cells, M9 minimal medium was introduced using a syringe pump at shear rates of 0 s^−1^, 240 s^−1^, or 800 s^−1^. Images were taken every hour for 24 h. Using ImageJ software, we used a color threshold to generate a contour around the cells in each image, quantified the number of pixels within the contour for each frame, and divided that number by the total number of pixels in the image. We then reported the percentage of pixels covered by the bacterial cells as the percent area covered.

### Conditioned media

Conditioned media was prepared by using a 27 cm-channel microfluidic device preloaded with a high density (≥1 OD) of *P. aeruginosa* cells. M9 minimal medium with no added glucose was then flowed through the 27 cm channel at an 800 s^−1^ shear rate. The flow-through of the experiment was collected in a sterile 50 mL tube. After the collection of the flow-through was completed, the flow-through was filtered through a 0.22 µm filter Steriflip unit (MilliporeSigma). Conditioned media was then supplemented with glucose as necessary for individual experiments.

### Determination of carbon and nitrogen uptake rates for simulations

As uptake rates have not been empirically measured or published, we estimated them by varying these rates in the simulation to reproduce the no-flow experimental results of the manuscript. In detail, we simulated cells in a channel without flow, which resembles a test tube condition. From Fig. 2, we know the relation between available glucose/nitrogen concentration and the instantaneous cell growth rate. For a set uptake rate, we simulated bacterial growth in the channel over time and analyzed the maximum growth (max OD) as a function of initial glucose/nitrogen concentration and compared the result to our experimental results in Fig. 1. We then updated the uptake rates until we found the best match between simulation and experiment. The uptake rates that were determined in this manner were then used for flow simulations.

### Flow simulations

Flow simulations were performed as described previously (14). In brief, we combined the laminar transport due to flow with a Brownian dynamics simulation. Initially, molecules in the channel were seeded at random with the concentration *c*_initial_. Flow was modeled with a parabolic flow speed profile $v\left(y\right)=v_{0}\left(1-{\left(\frac{y-\frac{h}{2}}{\frac{h}{2}}\right)}^{2}\right)$ according to the Hagen-Poiseuille equation, with the channel height *h* and width *w*, maximum velocity $v_{0}=1.7\cdot 10^{-11}\frac{3Q}{2hw}$ in the center of the channel, and flow rate *Q* in mL/min. In timesteps of D*t* = 1 ms, each particle was allowed to move along the channel due to fluid flow according to its lateral position *v*(*y*). In addition, each particle was allowed to diffuse the distance $\frac{∆x/y}{∆t}=n_{0}\sqrt{\frac{24D}{∆t}}$ lateral to (*y*) and along (*x*) the channel, where *n*_0_ is a random number drawn from a uniform distribution in the interval −0.5 ≤ 𝑛_0_ ≤ 0.5 and diffusion coefficients $D_{glucose}=0.67\cdot 10^{-9\frac{m^{2}}{s}}$ (58) and $D_{NH4Cl}=2\cdot 10^{-9\frac{m^{2}}{s}}$(59).

Previous simulations assumed no change of particle uptake over time. Here, we remove this restriction and explicitly consider cell growth by updating the particle uptake rate based on the particle concentration in the environment during every time loop of the simulation. In brief, we used the experimentally determined growth rate *g*(*c*) as a function of glucose concentration *c*(*x*) along the channel and the fit function 𝑔(𝑥) = 𝐴𝑐(𝑥)𝑏/(𝑐(𝑥)𝑏 + 𝑐0𝑏) with the maximum cell division rate of A = 0.535 per hour, the concentration *c*0 = 0.033 at which g = A/2, and the exponent b = 1.0793. From this, the removal rate r(x, t + Δt) = r(x, t) * (1 + g(x) * Δt) and cell density d(x, t + Δt) = d(x, t) * (1 + g(x) * Δt) along the channel was updated during every simulation step with the time interval Δ*t*.

## SUPPLEMENTAL MATERIAL

Supplemental Material mbio.03446-25-s0001.docx Fig. S1 to S8.

ASM does not own the copyrights to Supplemental Material that may be linked to, or accessed through, an article. The authors have granted ASM a non-exclusive, world-wide license to publish the Supplemental Material files. Please contact the corresponding author directly for reuse.
