# Supplementary material for: Shear flow promotes bacterial growth and shapes spatial gradients by rapidly replenishing scarce nutrients
Source: mBio. 2026 Feb 27;17(4):e03446-25. doi: 10.1128/mbio.03446-25 (PMC13059767; doi:10.1128/mbio.03446-25)
Supplement: Supplemental Material — Fig. S1 to S8. [file mbio.03446-25-s0001.docx]

Supplementary Information for

**Shear flow promotes bacterial growth and shapes spatial gradients by rapidly replenishing scarce nutrients**

Gilberto C. Padron^1^, Sizhe Chen^1^, Anuradha Sharma^1^, Zil Modi^2^, Matthias D. Koch^2^, Joseph E. Sanfilippo^1^*

^1^Department of Biochemistry, University of Illinois at Urbana-Champaign, Urbana, IL 61801

^2^Department of Biology, Texas A&M University, College Station, TX 77843

*To whom correspondence should be addressed. Email: josephes@illinois.edu

**This includes:**

Supplemental Figures S1 to S8


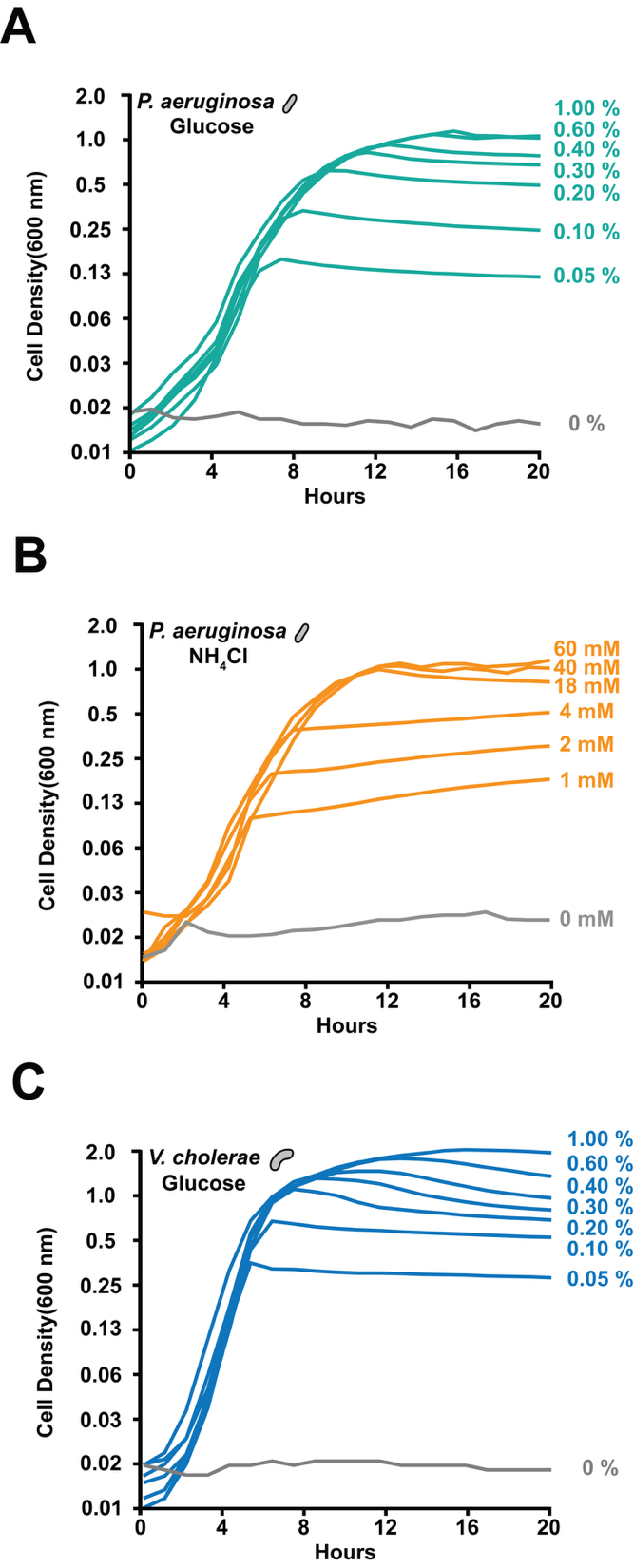


**Figure S1. Increasing nutrient concentration does not impact growth rate.** Growth curves of *P. aeruginosa* cells in M9 minimal medium plotted on a logarithmic scale with varying glucose **(A)** and ammonium chloride **(B)** concentrations. **(C)** Growth curves of *V. cholerae* cells in M9 minimal medium plotted on a logarithmic scale with varying glucose concentrations. Cell density measured by optical density at 600 nm. While increasing glucose impacted maximal culture density, it did not impact growth rate.


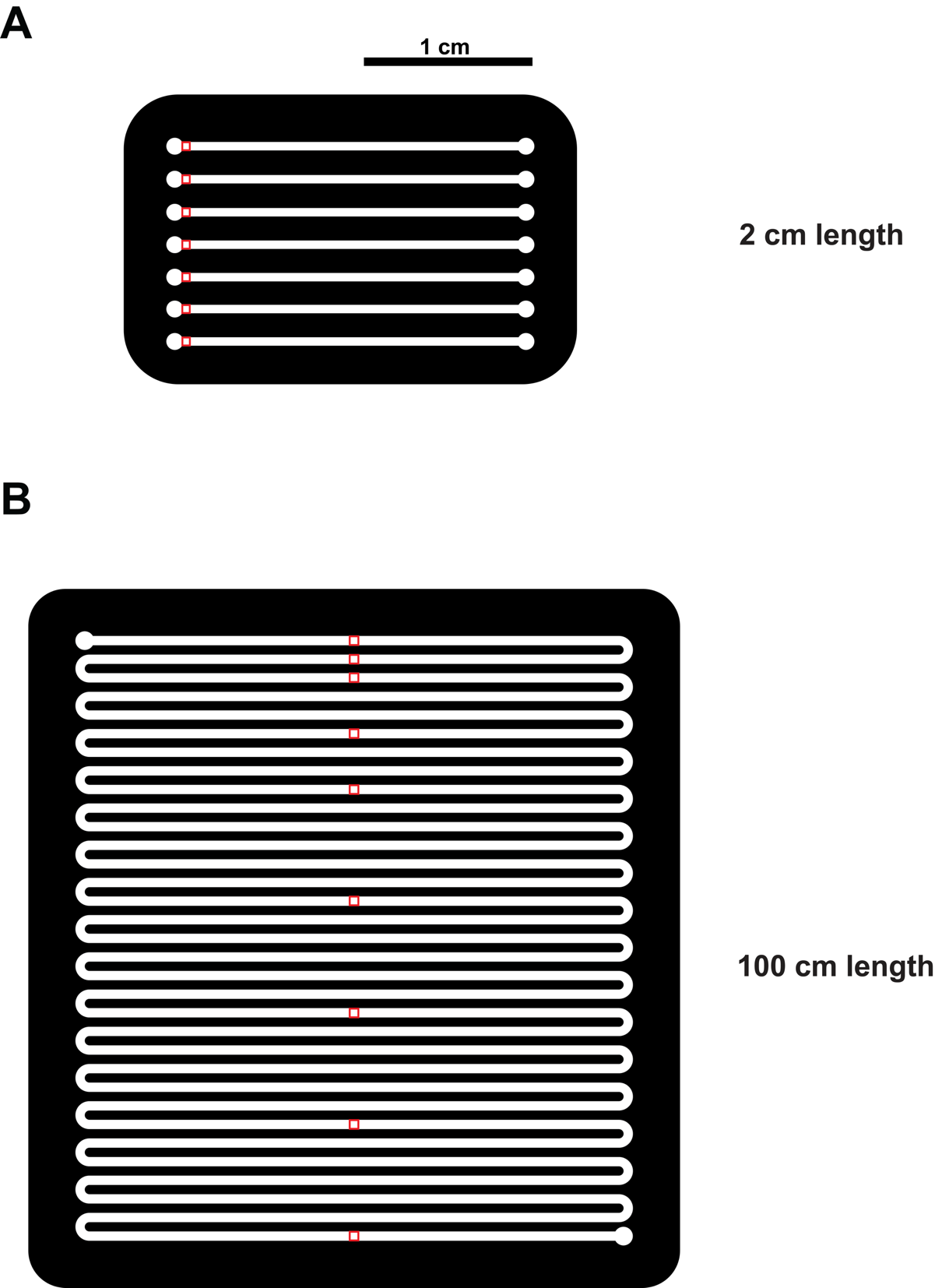


**Figure S2. Microfluidic devices used in this study. (A)** Top-down view of a 2 cm long device with 7 channels. **(B)** Top-down view of a 100 cm long device containing 32 turns. Red squares represent imaging locations. Scale bar is 1 cm. Figure and descriptive text were replicated and adapted from (17).


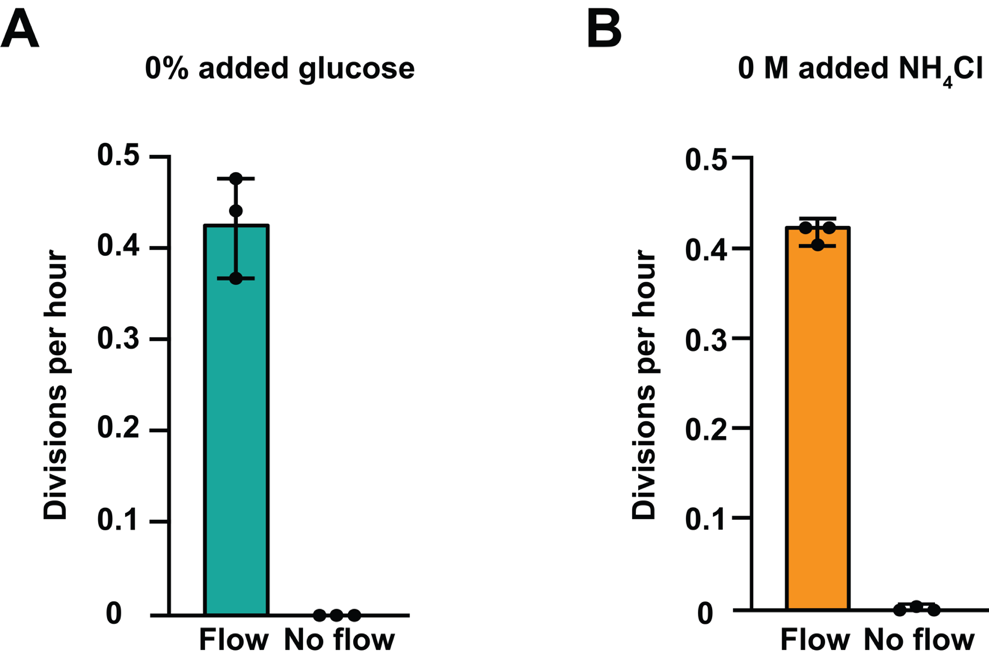


**Figure S3. Contaminant levels of carbon and nitrogen result in flow-dependent growth.**

Quantification of *Pseudomonas aeruginosa* growth in flow (800 sec^-1^) and no flow with no added glucose **(A)** or no added ammonium chloride **(B)** in M9 minimal medium over 8 hours. Quantification indicates the average and standard deviation of 3 biological replicates. For each biological replicate, 30 cells were chosen at random for quantification.


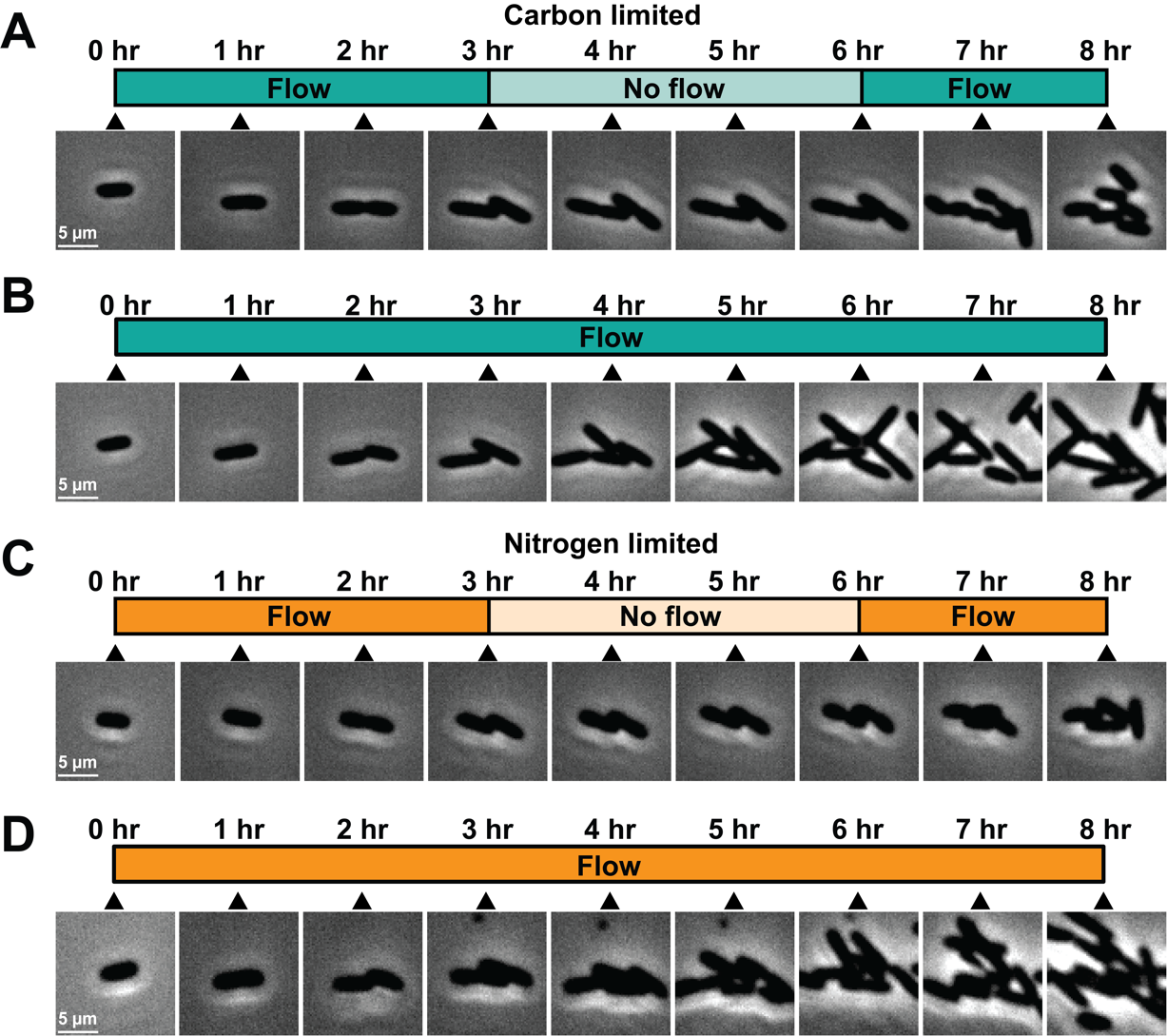


**Figure S4. Flow sustains bacterial growth under nutrient limited conditions.** Timeline and phase contrast images of *P. aeruginosa* under variable flow **(A)** or constant flow **(B)** in carbon limited conditions**,** and variable flow **(C)** or constant flow **(D)** in nitrogen limited conditions. When enabled, flow was set to a shear rate of 800 sec^-1^. Triangles indicate the times at which images were taken. Scale bar on images indicates 5 µm. Carbon limited indicates M9 minimal medium without an added carbon source and nitrogen limited indicates M9 minimal medium without an added nitrogen source. Triangles indicate the time at which image was taken.


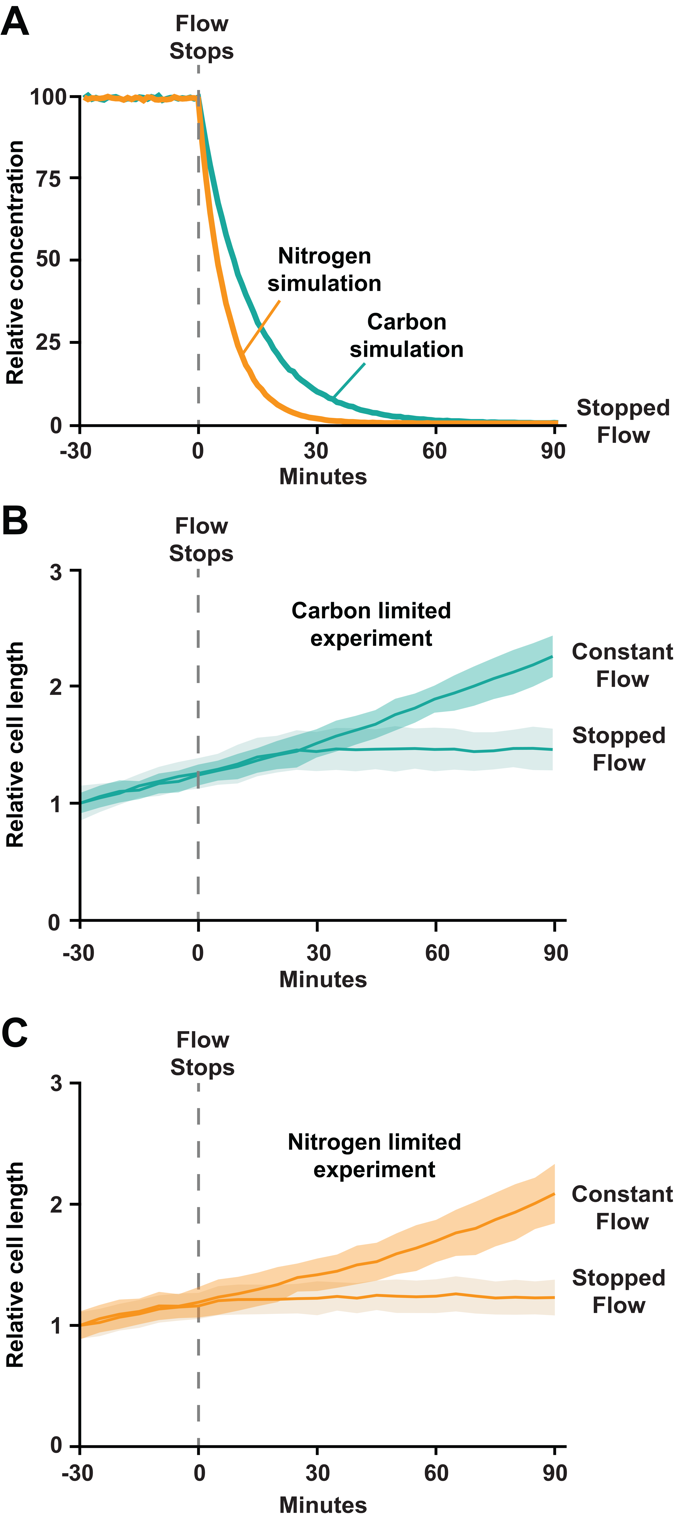


**Figure S5. *P. aeruginosa* rapidly depletes nutrients and stop growing when flow stops.** **(A)** Simulation displaying relative concentration of nutrients before and after flow is shut off in a channel seeded with *P. aeruginosa* cells. Teal line represents carbon limited simulation and orange line represents nitrogen limited simulation. Quantification of relative cell length (a measure of growth) during carbon limited **(B)** and nitrogen limited **(C)** experiments under constant flow and flow that was stopped. Constant flow indicates flow throughout the experiment, while stopped flow indicates flow that was stopped at the gray dashed line. When flow stops, cells stop growing in less than 30 minutes, which corresponds to the simulated time of nutrient depletion. Lines and shading show the average and standard deviation of three biological replicates. For each biological replicate, 30 cells were chosen at random for quantification.


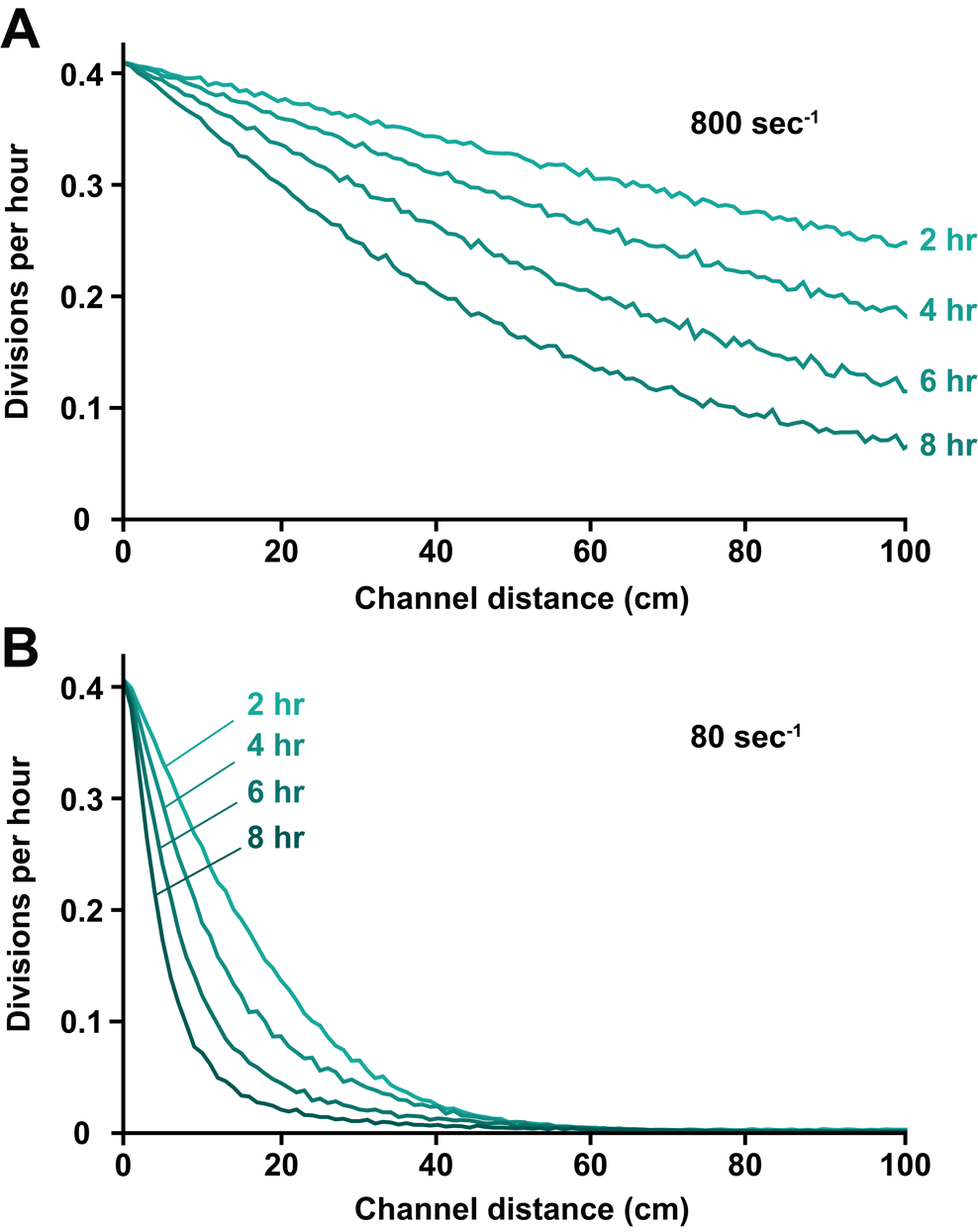


**Figure S6. Simulations predict that spatial gradients shift over time.** Simulations at 800 sec^-1^ **(A)** and 80 sec^-1^ **(B)** shear rates demonstrating how growth over time impacts spatial growth gradients. As cells grow, their collective ability to remove nutrients increases. In the simulations, a feedback was included where removal increases over time to represent the increase in biomass. Each line represents the rate of growth in divisions per hour at 2 hour time increments. 800 sec^-1^ shear rate generates longer gradients than 80 sec^-1^ because nutrients are delivered more quickly.


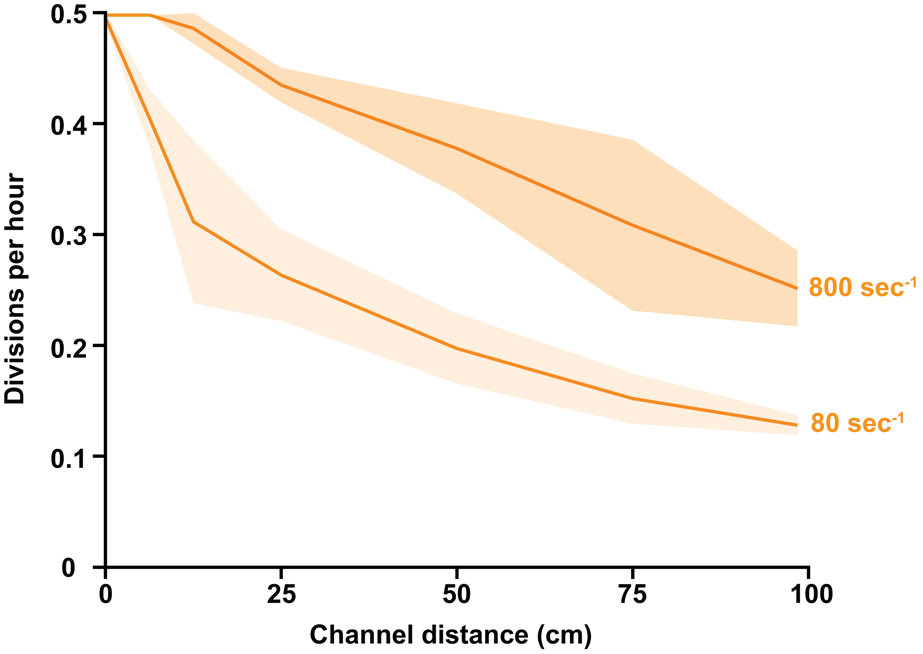


**Figure S7. Flow shapes gradients of nitrogen availability and growth**. Cell divisions per hour under nitrogen limited conditions across a 1 meter channel at different shear rates. For these experiments, M9 minimal medium was used with no added nitrogen source. Lines and shading represent the average of three biological replicates and standard deviation. For each biological replicate, 30 cells were chosen at random for quantification.


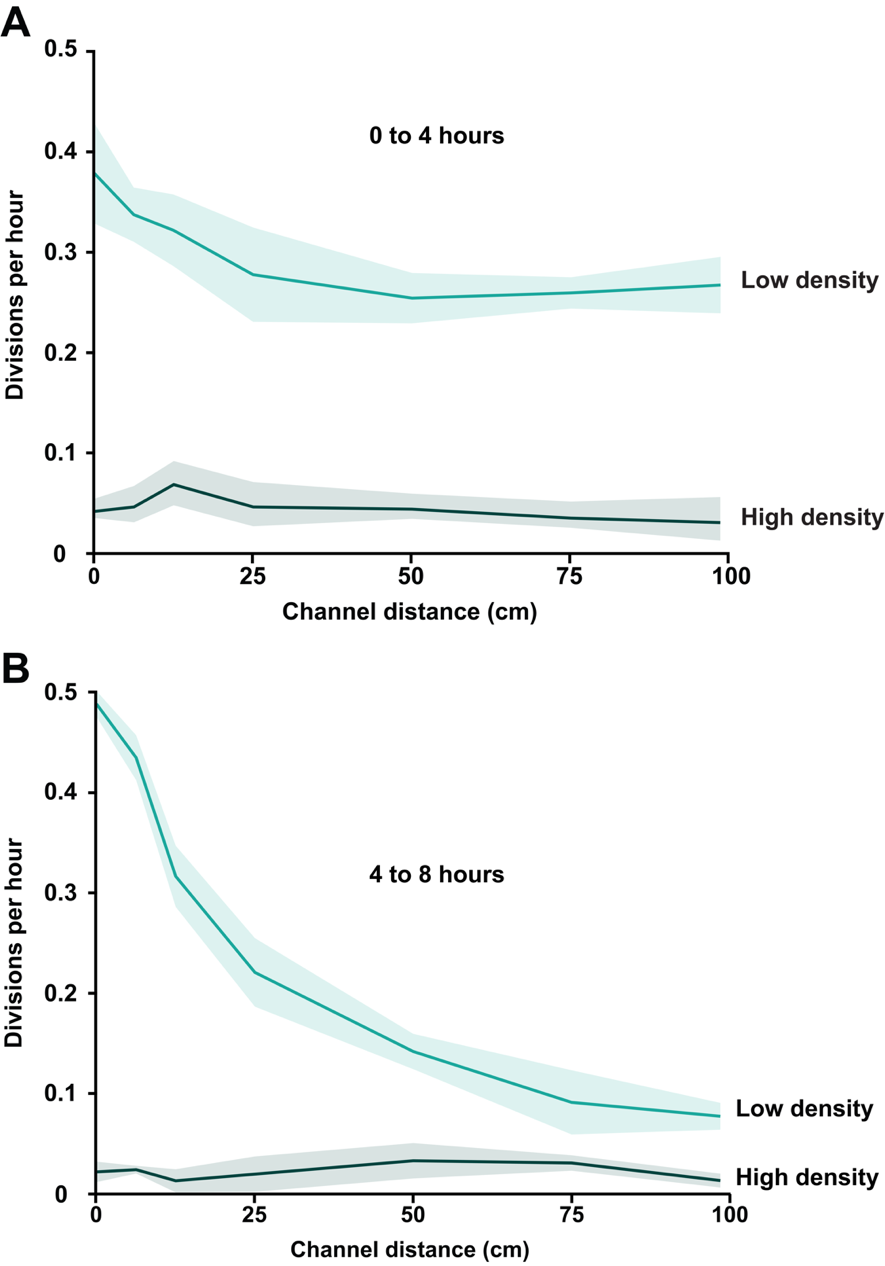


**Figure S8. Cell density impacts growth and shifts growth gradients over time.**

Quantification of cell divisions per hour in the first 4 hours **(A)** and second 4 hours **(B)** of an 8 hour carbon limited experiment at both low (10x dilution of a mid-log culture) and high cell density (10x concentrated version of a mid-log culture) at a shear rate of 800 sec^-1^. For these experiments, M9 minimal medium was used with no added carbon source. Lines and shading represent the average of three biological replicates and standard deviation. For each biological replicate, 30 cells were chosen at random for quantification.
